# Real‐World Outcomes of Immune Checkpoint Inhibitors in Head and Neck Squamous Cell Carcinoma: Analyzing Patient‐Specific Factors Influencing Survival and Response Rate

**DOI:** 10.1002/cnr2.70369

**Published:** 2025-11-11

**Authors:** Lena Huber, Constanze Kohler, Anne Lammert, Claudia Scherl, Sonja Ludwig, Annette Affolter, Nicole Rotter, Frederic Jungbauer

**Affiliations:** ^1^ Department of Otolaryngology, Head and Neck Surgery University Medical Center Mannheim, Heidelberg University Germany; ^2^ Medical Faculty Mainz University Germany

**Keywords:** head and neck squamous cell carcinoma, immune checkpoint inhibitors, immunotherapy response, nivolumab, pembrolizumab, predictive biomarkers, progression‐free survival, real‐world outcomes, survival analysis

## Abstract

**Background:**

Head and neck squamous cell carcinoma (HNSCC) is a globally significant disease with poor survival outcomes. Immune checkpoint inhibitors (ICIs) such as pembrolizumab and nivolumab have improved treatment paradigms, yet their real‐world efficacy and the factors influencing treatment outcomes remain underexplored.

**Aims:**

This study aimed to evaluate real‐world outcomes of pembrolizumab and nivolumab therapy in patients with HNSCC and to identify clinical and laboratory factors associated with overall survival (OS), progression‐free survival (PFS), and objective response rate (ORR).

**Methods and Results:**

We conducted a retrospective analysis of 45 HNSCC patients treated with pembrolizumab or nivolumab at the University Medical Center Mannheim. Patient‐specific factors, including tumor characteristics, PD‐L1 expression, and laboratory parameters, were assessed using Kaplan–Meier estimation, log‐rank tests, and multivariate regression models. The median OS and PFS were 10.4 months and 7.4 months, respectively, with an ORR of 22%. A tumor proportion score (TPS) ≥ 50% and absence of smoking or alcohol abuse significantly improved ORR, while female sex, high neutrophil‐to‐lymphocyte ratio (NLR), and elevated leukocyte counts were associated with inferior OS and PFS. Real‐world outcomes largely aligned with the pivotal trials Keynote‐048 and CheckMate 141.

**Conclusion:**

This study underscores the predictive value of TPS and patient lifestyle factors in ICI treatment for HNSCC. The findings also highlight sex‐specific differences, as well as NLR and leukocyte count as potential prognostic factors. Larger, more diverse cohorts are needed to confirm these results and refine patient selection strategies.

## 1 Introduction

Head and neck squamous cell carcinoma (HNSCC) presents a significant global health challenge, characterized by its aggressive nature and poor survival rates. The approval of immune checkpoint inhibitors (ICIs), notably pembrolizumab and nivolumab, has revolutionized the standard of care for patients with recurrent or metastatic HNSCC.

The pivotal trials, Keynote‐048 [1] and CheckMate 141 [2] have demonstrated the efficacy of pembrolizumab and nivolumab, respectively, in improving overall survival (OS) and progression‐free survival (PFS) among patients with recurrent and metastatic HNSCC (r/m HNSCC). However, these studies often involve selected patient populations under controlled conditions, which may not fully represent the broader, more diverse patient demographics encountered in routine clinical practice. Consequently, there is a growing need to understand how these therapies perform in real‐world settings, where patient variability is greater. Notably, while ICIs have shown promise in prolonging survival, the objective response rate (ORR) remains low, underscoring the need to better understand the factors influencing treatment response. Patient‐specific variables, including performance status, tumor characteristics, and biomarkers such as PD‐L1 expression, may profoundly affect ORR and other clinical outcomes.

This study aims to address these gaps by analyzing real‐world data on patients treated with pembrolizumab and nivolumab for HNSCC. We focus on elucidating the relationship between individual patient factors and key clinical outcomes OS, PFS, and ORR. In addition to reporting treatment outcomes in our cohort, we aim to evaluate whether clinical and laboratory parameters previously described in the literature as potential predictive or prognostic markers for ICI therapy in HNSCC can be confirmed in our patient population. This exploratory validation was intended to place our findings in the context of existing evidence and to contribute to the identification of factors relevant for patient selection in clinical practice. By leveraging real‐world evidence, our goal is to provide actionable insights that can optimize patient selection and management strategies, ultimately improving outcomes in this heterogeneous disease.

## 2 Methods

A structured literature review was performed to identify clinical and laboratory parameters associated with outcomes of immune checkpoint inhibitor (ICI) therapy across cancer types. We searched PubMed/MEDLINE in December 2023 using combinations of the following terms: “immunotherapy” OR “immune checkpoint inhibitor” OR “PD‐1” OR “PD‐L1” AND “predictive marker” OR “predictor” OR “prognostic factor” OR “biomarker” OR “marker.” The search was limited to articles published in English between 2010 and 2023. Titles and abstracts were screened to identify relevant studies, and full texts were assessed for eligibility. Both prospective clinical trials and retrospective cohort studies were included if they reported an association between clinical or laboratory parameters and treatment outcomes such as response, progression‐free survival, or overall survival under ICI therapy. References from key articles were manually reviewed to identify additional relevant publications. Although our study focuses on HNSCC, the literature review considered all tumor types treated with ICIs to ensure a comprehensive overview of potential prognostic and predictive markers.

The results of the literature research were that clinical factors (ECOG performance status, age, sex, primary tumor site, HPV/p16 status, alcohol/tobacco abuse history, prior therapies, tumor stage, disease status, body mass index (BMI)), laboratory parameters (neutrophil‐to‐lymphocyte ratio, platelet‐to‐lymphocyte ratio, absolute lymphocyte count, albumin, lactate dehydrogenase (LDH), C‐reactive protein (CRP), Glasgow prognostic score (GPS), CRP to albumin ratio, hemoglobin, lymphocyte‐to‐monocyte ratio), tumor and molecular markers (PD‐L1 expression, tumor mutational burden, immune gene signatures), and treatment‐related variables (type of ICI, monotherapy versus chemo‐immunotherapy, and occurrence of immune‐related adverse events (irAE)) were reported as predictors of immunotherapy outcomes in solid tumors. We concentrated on the most consistently mentioned factors in HNSCC and therefore analyzed all of the above except for the following: C‐reactive protein to albumin ratio, hemoglobin, and lymphocyte‐to‐monocyte ratio were not included due to limited relevance. Subgroup analyses by type of ICI and analyses of chemo‐immunotherapy were not performed because of the small sample size, and tumor mutational burden and immune gene signatures were not included as they could not be collected retrospectively [3, 4]. All laboratory parameters were collected at the time of the first administration of ICI. Parameters with established reference ranges were categorized as within or outside the normal range, with the respective reference values provided in Table 1. For neutrophil‐to‐lymphocyte ratio (NLR) and platelet‐to‐lymphocyte ratio (PLR), cut‐offs of 5 and 200, respectively, were applied, as these are the most commonly reported thresholds in the literature and facilitate comparison with previous studies [4]. p16 status was collected as a surrogate marker for HPV status because it was consistently available in our cohort. IrAEs were categorized in Grades 1–5 according to the Common Terminology Criteria for Adverse Events (v 5.0, 2017). For statistical analysis, irAEs were categorized as grade 1 versus grade ≥ 2, as higher‐grade events generally require treatment interruption, immunosuppressive therapy, or permanent discontinuation of ICI, and are therefore clinically more relevant. PD‐L1 expression status (combined positive score (CPS) and tumor proportion score (TPS)) could not be collected in three patients receiving nivolumab. Three out of 45 values (6.7%) were missing and identified as missing completely at random (MCAR). Due to the low proportion and random pattern, mean imputation was applied. This method is acceptable in such cases to preserve sample size with minimal bias [5].

**TABLE 1:** Baseline characteristics of the study population.

	Total		Pembro.		Nivo.	
Patient characteristics						
*n*	45	100%	29	64%	16	36%
Sex						
Male	34	76%	22	49%	12	27%
Female	11	24%	7	16%	4	9%
Age	65	(44–85)	66	(47–85)	63.5	(44–81)
BMI	22.1	(17.2–35.7)	22.1	(17.2–28.7)	22.6	(18–35.7)
ECOG						
0	6	13%	3	7%	3	7%
1	24	53%	17	38%	7	16%
2	11	24%	6	13%	5	11%
3	2	4%	1	2%	1	2%
4	2	4%	2	4%	0	0%
Alcohol/smoking						
None	11	24%	7	16%	4	9%
Smoking	11	24%	5	11%	6	13%
Alcohol	4	9%	4	9%	0	0%
Both	19	42%	13	29%	6	13%
Prior treatment						
RCT	31	69%	17	38%	14	31%
RT	11	24%	9	20%	2	4%
None	3	7%	3	7%	0	0%
Tumor characteristics						
TPS						
≥ 50%	4	9%	4	9%	0	0%
< 50%	38	84%	25	56%	13	29%
Unknown	3	7%	0	0%	3	7%
CPS						
≥ 1	30	67%	28	62%	2	4%
≥ 20	7	16%	7	16%	0	0%
Unknown	3	7%	0	0%	3	7%
Stage						
2	4	9%	3	7%	1	2%
3	1	2%	1	2%	0	0%
4	40	89%	25	56%	15	33%
p16 positive	11	24%	8	18%	3	7%
Primary tumor						
Parotid gland	1	2%	0	0%	1	2%
CUP	1	2%	0	0%	1	2%
Nasopharynx	1	2%	1	2%	0	0%
Nose/sinuses	2	4%	0	0%	2	4%
Hypopharynx	7	16%	5	11%	2	4%
Larynx	4	9%	3	7%	1	2%
Oral cavity	4	9%	3	7%	1	2%
Oropharynx	25	56%	17	38%	8	18%
Disease status						
Local recurrence	9	20%	6	13%	3	7%
Residual tumor	8	18%	7	16%	1	2%
Metastatic	28	62%	16	36%	12	27%
Laboratory parameters						
Leukocyte count						
In norm (4.2–10.2/nL)	33	73%	19	42%	14	31%
Below norm	6	13%	5	11%	1	2%
Above norm	6	13%	5	11%	1	2%
Lymphocyte count						
In norm (1.4–3.2/nL)	3	7%	2	4%	1	2%
Below norm	38	84%	24	53%	14	31%
Above norm	4	9%	3	7%	1	2%
CRP						
In norm (< 5 mg/L)	12	27%	10	22%	2	4%
Above norm	33	73%	19	42%	14	31%
Albumin						
In norm (34–50 g/L)	19	42%	15	33%	4	9%
Below norm	26	58%	14	31%	12	27%
LDH						
In norm (109–250 U/L)	31	69%	21	47%	10	22%
Above norm	14	31%	8	18%	6	13%
GPS						
0	10	22%	8	18%	2	4%
1	15	33%	11	24%	4	9%
2	20	44%	10	22%	10	22%
PLR						
Median (range)	393.9	(108–1333.3)	393.9	(108–874.1)	372.8	(132–1333.3)
> 5	29	64%	18	40%	11	24%
NLR						
Median (range)	6.8	(2.5–23.7)	6.8	(2.5–10.9)	6.7	(3.5–23.7)
> 200	37	82%	23	51%	14	31%

*Note:* Data are *n* and % or median (range).

Abbreviations: BMI = body mass index; CPS = combined positive score; CRP = C‐reactive protein; ECOG = Eastern Cooperative Oncology Group performance scale; GPS = Glasgow prognostic score; LDH = lactate dehydrogenase; nivo. = nivolumab; NLR = neutrophil‐to‐lymphocyte ratio; pembro. = pembrolizumab; PLR = platelet‐to‐lymphocyte ratio; TPS = tumor proportion score.

The patient data were collected at the department of otolaryngology, head and neck surgery, University Medical Center Mannheim, Heidelberg University. All 45 patients who received ICI monotherapy with pembrolizumab or nivolumab for the treatment of HNSCC between May 2018 and March 2024 were included. All treatment decisions were made by our multidisciplinary tumor board in accordance with the approved indications for pembrolizumab/nivolumab as well as NCCN and German clinical guidelines. Patients without prior chemotherapy and with a CPS ≥ 1 received pembrolizumab. In cases where patients were eligible for both agents, the final decision was made by the treating physician, typically in consultation with the patient. Most patients with a CPS ≥ 1 received pembrolizumab, unless other considerations—such as patient preference or the dosing schedule (biweekly vs. triweekly)—favored the use of nivolumab. Pembrolizumab regimen was 200 mg every 3 weeks and nivolumab regimen was 240 mg every 2 weeks, with treatment continued until disease progression or unacceptable toxicity. Data collection ended on March 15, 2024. Patients had follow‐up appointments every 2 months in the first year, every 3 months in the second year, every 4 months in the third year, every 6 months in the fourth year and annually from the fifth year onwards. Patients were contacted if they missed follow‐up appointments and all follow‐ups were conducted ±2 weeks until the end of data collection or death. One patient receiving nivolumab had a primary tumor of the parotid gland but was included in the study because it was a p16 positive SCC. Since ORR and OS differed between the pembrolizumab and pembrolizumab + chemotherapy groups in the pivotal Keynote‐048 trial, combination therapies were not included in this study. Patients who received off‐label ICIs as part of a clinical trial were excluded. This retrospective single‐center study was approved by the ethics committee of the University of Heidelberg (2024‐807).

OS and PFS were defined as the time from diagnosis of recurrent or metastatic HNSCC to death from any cause (OS) or disease progression/death from any cause (PFS). ORR was determined by analyzing the first computer tomography (CT) staging after 12 weeks using the (Response Evaluation Criteria in Solid Tumours in Cancer Immunotherapy Trials) iRECIST guidelines [6]. Results were categorized into progressive disease (PD), partial response (PR), stable disease (SD), and complete response (CR). ORR was defined as the proportion of patients showing either PR or CR in the first staging. Patients with SD continued treatment with ICI, while those with PD were switched to an alternative therapy.

Statistical analyses were performed using SPSS Statistics (v29.0.2.0). Median follow‐up was calculated with the reverse Kaplan–Meier method. ORR was analyzed using univariate logistic regression. Variables with *p* ≤ 0.05 were entered simultaneously into a multivariate logistic regression model using the enter method. For each variable, odds ratios (ORs) with corresponding *p*‐values were reported. OS and PFS were estimated using the Kaplan–Meier method, with censoring applied to patients alive at the end of follow‐up. To identify predictors of OS and PFS, we used the log‐rank (Mantel‐Cox) test, which yields a chi‐square statistic, and reported the corresponding *p*‐values. Variables with *p* ≤ 0.05 in the log‐rank test were entered simultaneously into multivariate Cox proportional hazards models using the enter method. Adjusted hazard ratios (HRs) with 95% confidence intervals were reported. Statistical dependencies between the parameters were identified and eliminated using Pearson correlation analysis (|*r*| > 0.5). No correction for multiple testing was applied, as all univariate analyses were exploratory and used exclusively for guiding variable selection.

## 3 Results

### 3.1 Patient, Tumor and Treatment Characteristics

A total of 45 patients were included in the study, of whom 29 received pembrolizumab and 16 received nivolumab. Since pembrolizumab is only approved for the treatment of HNSCC in patients with CPS ≥ 1, all patients with CPS < 1 or unknown PD‐L1 expression were treated with nivolumab. At the first staging assessment, stable disease was observed in 17 patients (38%), progressive disease in 18 patients (40%), and a partial response in 10 patients (22%). No complete response was observed at the first staging. Baseline characteristics are summarized in Table 1.

By the end of data collection, 40 patients had discontinued ICI therapy (88.9%). Nine patients had received at least one subsequent anticancer therapy (Table 2), and eight had undergone palliative radiotherapy for pain or other symptom relief, with some receiving both. The other 30 patients either continued ICI until death/end of data collection or received no additional treatment after the discontinuation of ICI. Among the 19 patients who discontinued ICI without further treatment, 11 patients experienced rapid disease progression, while five had a decline in general condition or comorbidities that precluded further treatment. Three patients discontinued treatment due to irAEs. At the time of data cutoff, 12 patients were still alive: five continued ICI therapy, three had received or were receiving subsequent therapies, one was switched to best supportive care due to tumor progression shortly before data cutoff, two maintained stable disease after the discontinuation of ICI, and one remained in complete remission after the discontinuation of ICI therapy.

**TABLE 2:** Subsequent anticancer therapy.

Anticancer therapy	Post‐nivolumab	Post‐pembrolizumab	All patients
Any^a^	2	7	9
Docetaxel	2	3	5
EGFR inhibitor	1	4	4
Gemcitabine		1	1
Platinum‐based	1	4	5
Pembrolizumab		1	1
5‐Fluorouracil	1	1	2

^a^ Patients may have received more than one subsequent anticancer therapy overall or of a specific category.

### 3.2 Immune‐Related Adverse Events

Immune‐related adverse events (irAEs) occurred in 10 patients (22%) undergoing ICI therapy. The irAEs were classified as grade 1 (*n* = 3), grade 2 (*n* = 4), and grade 3 (*n* = 3). Treatment was discontinued in three patients and temporarily paused in three patients. Among the 10 patients with irAEs, 8 did not experience disease progression until death or the end of data collection. All patients who developed grade 3 irAEs remained alive and progression‐free at the end of data collection, with treatment having been paused for more than 12 months. The reported irAEs in this cohort included pneumonitis, skin rash and pruritus, liver enzyme elevation, leukopenia, gastrointestinal events (diarrhea, gastroenteritis), and thyroiditis.

In the univariate analyses, the occurrence of immune‐related adverse events was significantly associated with improved PFS and OS; however, this association did not remain significant in the multivariate analysis.

### 3.3 Comparison of Collected Real‐World Data to Keynote‐048 [1] and CheckMate 141 [2]

In the nivolumab arm of the CheckMate 141 study, the median age was 59 (range 29–83), while in the Keynote‐048 study, it was 62 (IQR 56–68). In our cohort, the median age was 65 (44–85).

In terms of gender distribution, 82.1% of patients in the nivolumab arm of the CheckMate 141 study and 83% of patients in the pembrolizumab arm of the Keynote‐048 study were male. In our cohort, 76% of patients were male.

Regarding performance status, 99.2% of patients in the nivolumab arm of the CheckMate 141 study and 100% of those in the pembrolizumab arm of the Keynote‐048 study had an ECOG performance status of 0 or 1. In our cohort, 66% of patients had an ECOG performance status of 0 or 1 and 32% had an ECOG score of 2, 3, or 4.

In our overall study population, the median OS was 10.4 months (95% CI 0–22), PFS was 7.4 months (95% CI 4.3–10.5), and ORR was 22%. For subset analyses in alignment with Keynote‐048 and CheckMate 141, refer to Table 3. In both the Keynote‐048 and CheckMate 141 studies, only the Kaplan–Meier curves were provided, and raw data were not available, precluding direct statistical comparison of OS and PFS between studies. In most cases, overlapping confidence intervals indicate no significant difference. For PFS in patients receiving nivolumab, non‐overlapping confidence intervals and a clear median separation indicate a statistically significant difference.

**TABLE 3:** Comparison of our OS/PFS/ORR in our own data with the Keynote‐048 and CheckMate 141 studies.

	Keynote‐048 CheckMate 141	Own data
OS		
Pembro. CPS ≥ 1	12.3 (10.8–14.9)	17.7 (5.1–30.3)
Pembro. CPS ≥ 20	14.9 (11.6–21.5)	^a^
Nivolumab	7.5 (5.5–9.1)	7.8 (5–10.5)
PFS		
Pembro. CPS ≥ 1	3.2 (2.2–3.4)	8 (0–16.2)
Pembro. CPS ≥ 20	3.4 (3.2–3.8)	^a^
Nivolumab	2 (1.9–2.1)	5 (2.2–8)
ORR		
Pembro. CPS ≥ 1	19%	17%
Pembro. CPS ≥ 20	23%	43%
Nivolumab	13.3%	31%

*Note:* Data are Kaplan–Meier estimates of median (95% CI) PFS and OS in months or ORR in %.

Abbreviations: CPS = combined positive score; ORR = objective response rate; OS = overall survival; pembro. = pembrolizumab; PFS = progression‐free survival.

^a^ Kaplan–Meier estimates could not be calculated because 5/7 patients were still alive at the time of data collection.

In the pembrolizumab group of the original Keynote‐048 study, the median follow‐up was 11.5 months (IQR 5.1–20.8). In the updated five‐year analysis [7], the median follow‐up was 69.2 months (IQR 65.0–73.6), with a treatment discontinuation rate of 97.7% and a follow‐up rate of 99.7%. In the CheckMate 141 study, the initial report documented a median follow‐up of 5.1 months (range 0–16.8), while in the updated 2‐year analysis [8] the median follow‐up for the nivolumab monotherapy arm was 24.2 months (range 0.1–34.6), with a treatment discontinuation rate of 97.6% and a follow‐up rate of 99.6%. In our cohort, the median follow‐up was 35.1 months (IQR 21.2–63.7), with a treatment discontinuation rate of 88.9% and a follow‐up rate of 100%. With updated ascertainment as of September 19, 2025 (data unpublished), the treatment discontinuation rate remained 88.9% and the follow‐up rate 100%; the median follow‐up was 53.3 months (IQR 32.3–81.9 months).

### 3.4 Analysis of the Effect of Patient‐Specific Factors on ORR

Univariate analysis identified two factors with a significant positive impact on the objective response rate: the absence of alcohol and tobacco abuse (*p* = 0.033) and a TPS ≥ 50% (*p* = 0.008). No other factors approached statistical significance in the univariate analysis. Consequently, only these two factors were included in the subsequent multivariate analysis. In the multivariate logistic regression, the absence of alcohol and tobacco abuse (*p* = 0.031) and a TPS ≥ 50% (*p* = 0.021) were confirmed to be significantly associated with improved response rates. For further details, refer to Table 4.

**TABLE 4:** Univariate and multivariate analyses of the effect of patient‐specific factors on ORR.

	Univariate				Multivariate			
	*p*	OR	95% CI		*p*	OR	95% CI	
			Lower	Upper			Lower	Upper
No alcohol/smoking	0.033^a^	4.833	1.057	22.091	0.031^a^	6.548	1.189	36.059
TPS ≥ 50	0.008^a^	14.571	1.315	161.418	0.021^a^	21.107	1.600	278.477

Abbreviations: CI = confidence interval; OR = odds ratio; ORR = objective response rate; TPS = tumor proportion score.

^a^ Data with a significant influence on ORR with a *p*‐value ≤ 0.05.

### 3.5 Analysis of the Effect of Influential Patient‐Specific Factors on OS

**FIGURE 1:**
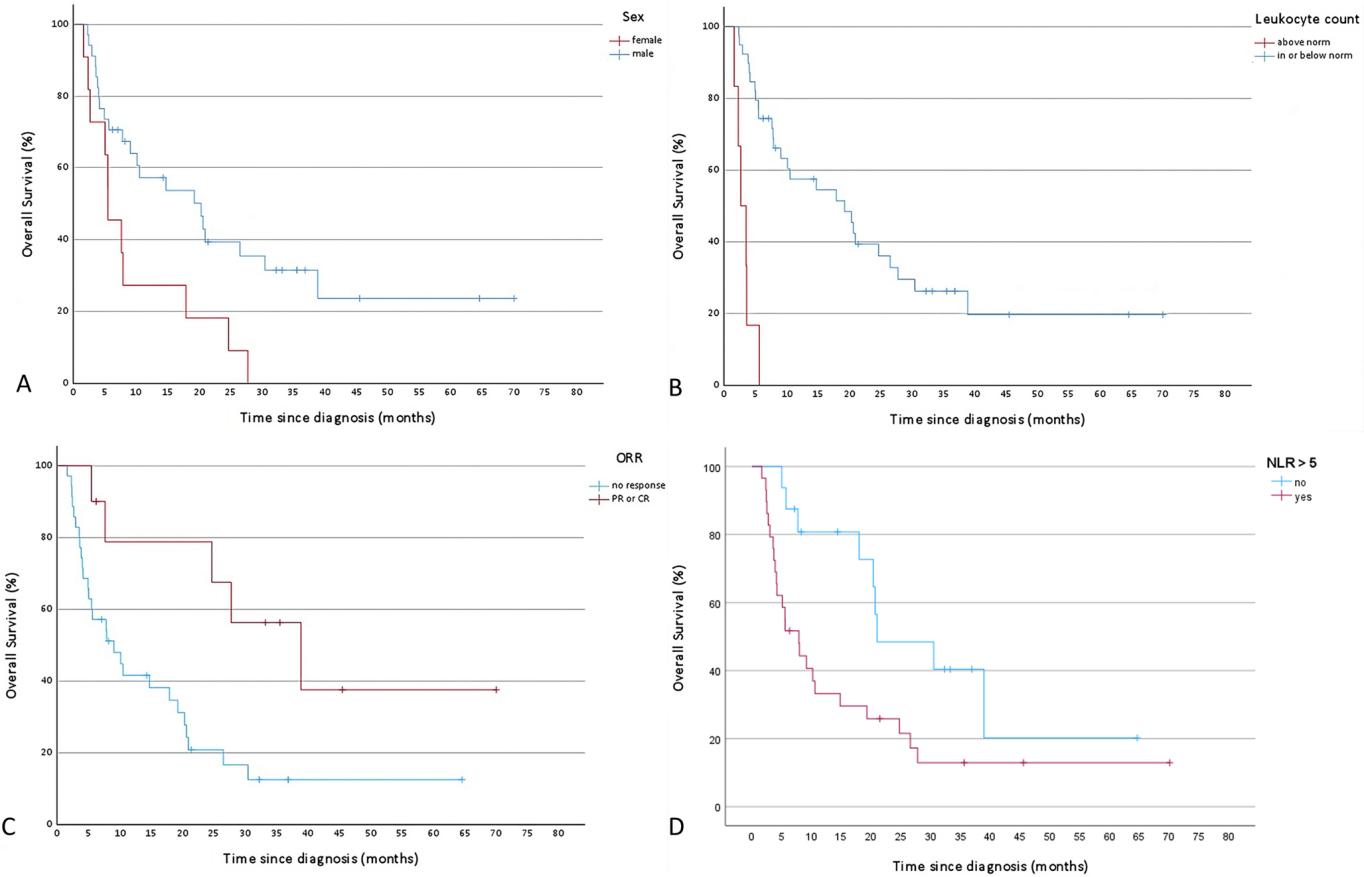
Kaplan–Meier estimates of overall survival stratified by (A) sex, (B) leukocyte count (C) overall response rate (ORR) and (D) neutrophil‐lymphocyte ratio (NLR). B–D were significant in multivariate analysis, A showed significance only in univariate analysis. Tick marks indicate censoring at the end of data collection. (A) Male patients (blue curve) show longer overall survival compared to female patients (red curve). (B) Patients with normal or low leukocyte counts (blue curve) show longer overall survival than patients with leukocytosis (leukocyte count above norm; red curve). (C) Responders to treatment—defined as achieving partial response (PR) or complete response (CR)—(red curve) show longer overall survival compared to non‐responders (blue curve). (D) Patients with NLR < 5 (blue curve) show longer overall survival than patients with NLR > 5.

The log‐rank test identified the following factors as significantly influencing OS: female sex (*p* = 0.011), age ≥ 80 (*p* = 0.031), ECOG > 1 (*p* = 0.025), ORR (*p* = 0.012), irAE ≥ 2 (*p* = 0.048), TPS ≥ 50% (*p* = 0.037), primary tumor in the oral cavity (*p* = 0.014), leukocyte count above the norm (*p* = 0.001), albumin in the norm (*p* = 0.014), GPS > 1 (*p* = 0.001) and NLR > 5 (*p* = 0.018). These factors were subsequently analyzed using a Cox proportional hazards model. The model confirmed the significant association of leukocyte count above the norm (*p* = 0.005, HR = 7.8) and NLR > 5 (*p* = 0.008, HR = 3.7) with worse OS and confirmed the association of ORR (*p* = 0.005, HR = 0.1) with improved OS. The Kaplan–Meier curves for the significant predictors in the multivariate analysis and for female sex are given in Figure 1. For additional data, refer to Table 5.

**TABLE 5:** Univariate and multivariate analyses of the effect of patient‐specific factors on OS.

	Univariate		Multivariate			
	*p*	*χ* ^2^	*p*	HR	95% CI	
					Lower	Upper
Female sex	0.011^a^	6.483	0.077	2.652	0.899	7.824
Age ≥ 80	0.031^a^	4.629	0.57	1.467	0.391	5.507
ECOG > 1	0.025^a^	5.042	0.551	0.703	0.293	1.926
ORR	0.012^a^	6.370	0.005^a^	0.138	0.035	0.546
irAE ≥ grade 2	0.048^a^	3.918	0.647	0.703	0.156	3.176
TPS ≥ 50	0.037^a^	4.329	0.759	0.683	0.06	7.803
Primary: oral cavity	0.037^a^	4.363	0.327	2.104	0.476	9.307
Leukocyte count above norm	0.001^a^	28.099	0.005^a^	7.795	1.866	32.569
Albumin in norm	0.014^a^	5.989	0.579	1.514	0.350	6.541
GPS > 1	0.001^a^	12.807	0.225	2.486	0.572	10.804
NLR > 5	0.018^a^	5.641	0.008^a^	3.736	1.42	9.829

Abbreviations: CI = confidence interval; ECOG = Eastern Cooperative Oncology Group performance scale; GPS = Glasgow prognostic score; HR = hazards ratio; NLR = neutrophil‐to‐lymphocyte ratio; OS = overall survival; ORR = objective response rate; TPS = tumor proportion score.

^a^ Data with a significant influence on OS with a *p*‐value ≤ 0.05.

### 3.6 Analysis of the Effect of Patient‐Specific Factors on PFS

**FIGURE 2:**
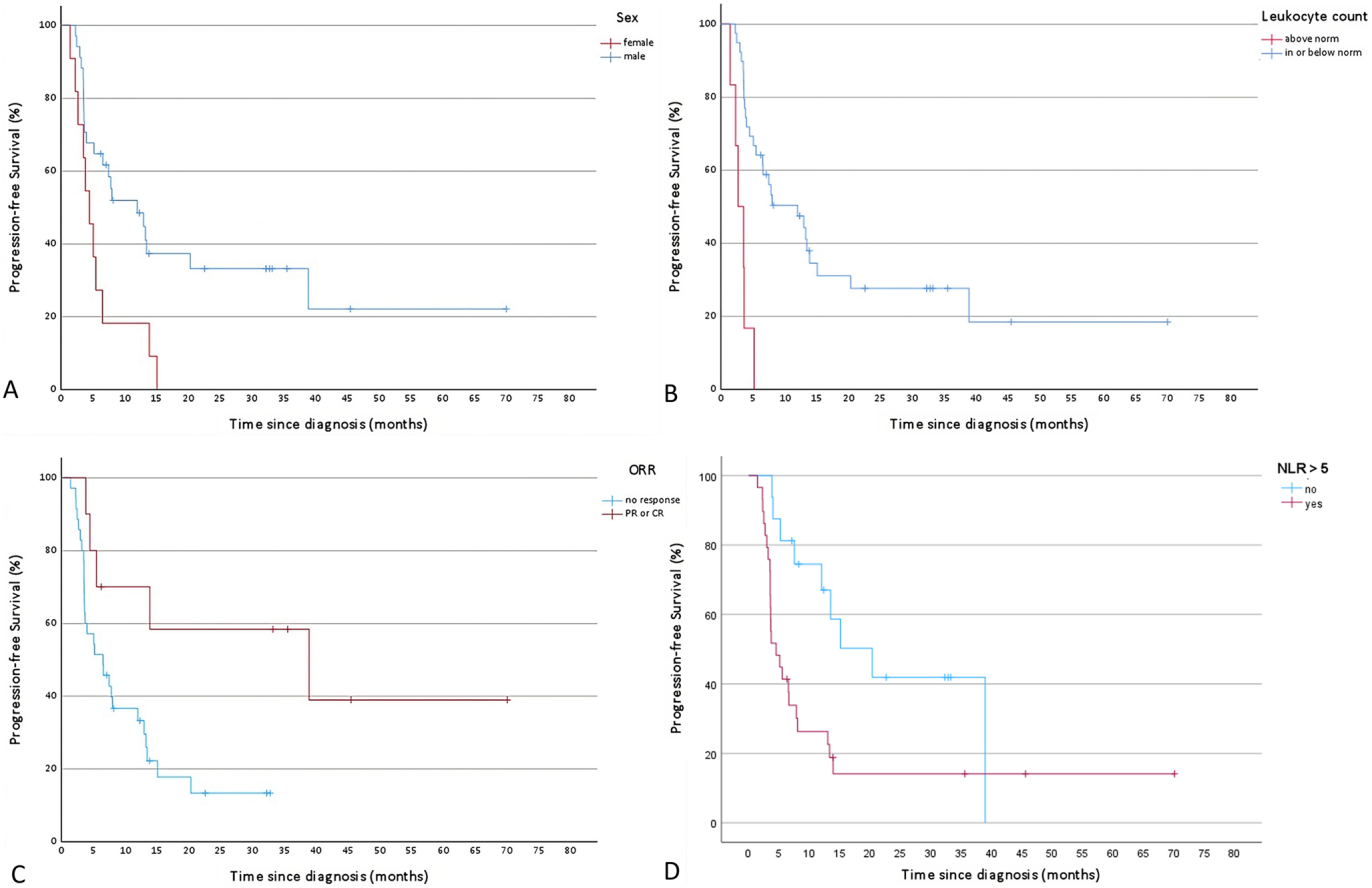
Kaplan–Meier estimates of progression‐free survival stratified by (A) sex, (B) leukocyte count (C) overall response rate (ORR) and (D) neutrophil‐lymphocyte‐ratio (NLR). (A)–(D) were significant in multivariate analysis. Tick marks indicate censoring at the end of data collection. (A) Male patients (blue curve) show longer progression‐free survival compared to female patients (red curve). (B) Patients with normal or low leukocyte counts (blue curve) show longer progression‐free survival than patients with leukocytosis (leukocyte count above norm; red curve). (C) Responders to treatment—defined as achieving partial response (PR) or complete response (CR)—(red curve) show longer progression‐free survival compared to non‐responders (blue curve). (D) Patients with NLR < 5 (blue curve) show longer progression‐free survival than patients with NLR > 5.

The log‐rank test identified the following factors as significantly influencing PFS: female sex (*p* = 0.008), ECOG > 1 (*p* = 0.016), ORR (*p* = 0.017), irAE ≥ 2 (*p* = 0.034), TPS ≥ 50% (*p* = 0.028), a leukocyte count above the norm (*p* = 0.001), CPS ≥ 20 (*p* = 0.044), CRP > 10 mg/L (*p* = 0.019), albumin in the norm (*p* = 0.003), GPS > 1 (*p* = 0.001), and NLR > 5 (*p* = 0.009). These factors were subsequently analyzed using a Cox proportional hazards model. The model confirmed the significant association of female sex (*p* = 0.021, HR = 2.7) and a leukocyte count above the norm (*p* = 0.01, HR = 4.5) and NLR > 5 (*p* = 0.018, HR = 3.4) with worse PFS and confirmed the association of ORR (*p* = 0.02, HR = 0.2) with improved PFS. The Kaplan–Meier curves for the significant predictors in the multivariate analysis are given in Figure 2. For additional data, refer to Table 6.

**TABLE 6:** Univariate and multivariate analyses of the effect of patient‐specific factors on PFS.

	Univariate		Multivariate			
	*p*	*χ* ^2^	*p*	HR	95% CI	
					Lower	Upper
Female sex	0.008^a^	6.960	0.021^a^	2.742	1.162	6.471
ECOG > 1	0.016^a^	5.831	0.904	1.056	0.433	2.577
ORR	0.017^a^	5.667	0.020^a^	0.213	0.058	0.781
irAE≥grade 2	0.034^a^	4.519	0.494	0.617	0.154	2.467
TPS ≥ 50	0.028^a^	4.816	0.422	0.339	0.024	4.746
CPS ≥ 20	0.044^a^	4.050	0.207	0.329	0.059	1.851
Leukocyte count above norm	0.001^a^	17.959	0.01^a^	4.465	1.431	13.926
CRP > 10 mg/L	0.019^a^	5.540	0.808	1.179	0.313	4.432
Albumin in norm	0.003^a^	9.060	0.671	1.376	0.316	6.001
GPS > 1	0.001^a^	19.772	0.434	2.167	0.312	15.058
NLR > 5	0.009^a^	6.773	0.018^a^	3.413	1.235	9.43

Abbreviations: CI = confidence interval; ECOG = Eastern Cooperative Oncology Group performance scale; GPS = Glasgow prognostic score; HR = hazards ratio; NLR = neutrophil‐to‐lymphocyte ratio; ORR = objective response rate; PFS = progression free survival; TPS = tumor proportion score.

^a^ Data with a significant influence on PFS with a *p*‐value ≤ 0.05.

## 4 Discussion

Our data represent a real‐world cohort of HNSCC patients treated with nivolumab or pembrolizumab monotherapy. Compared to Keynote‐048 and CheckMate 141 studies, our cohort was older, included a higher proportion of female patients, and had a greater prevalence of higher ECOG performance status scores. Although higher ECOG scores were significantly associated with worse OS and PFS in univariate analyses, these findings were not confirmed in the multivariate analysis. Consistent with our results, other real‐world data also demonstrate a correlation between higher ECOG scores and poorer OS [9, 10].

The OS and PFS observed in our cohort were similar to those reported in the Keynote‐048 and CheckMate 141 studies. However, the PFS for patients receiving Nivolumab in our cohort was notably longer (5 months) than the 2 months reported in the CheckMate 141 study. While this could be attributed to the longer follow‐up period in our study, even the updated CheckMate 141 analysis with a minimum follow‐up of 24.2 months reported a PFS of approximately 2 months [8]. This might be due to the earlier and more frequent CT stagings in the original studies (first staging at 9 weeks, then every 6 weeks). A direct statistical comparison of OS and PFS between studies is not possible due to the lack of raw data.

The objective response rate for patients receiving pembrolizumab with CPS ≥ 1 in our cohort was comparable to the Keynote‐048 study. Interestingly, the response rate for patients with CPS ≥ 20 receiving pembrolizumab and for those in the nivolumab group was approximately twice as high as reported in the original studies (Table 3). Nivolumab is approved for use in HNSCC irrespective of PD‐L1 status. A 2‐year long‐term survival update of the CheckMate 141 study analyzed OS by PD‐L1 expression [8] and confirmed that PD‐L1 expressors and non‐expressors derive a survival benefit from nivolumab, although only TPS ≥ 1% was considered, without reference to CPS. Consistent with previous findings, the patients in our cohort receiving nivolumab—most of whom had no or very low PD‐L1 expression—had an ORR of 32%, suggesting that response to nivolumab is independent of PD‐L1 expression. Similar trends of higher ORRs in real‐world settings have been reported elsewhere [9, 11].

Following the publication of the original data from the Keynote‐048 and CheckMate 141 trials, updated results have been reported with 4‐ and 5‐year follow‐up for Keynote‐048 [11, 12] and two‐year follow‐up for CheckMate 141 [8], which confirmed the OS and PFS findings of the initial analyses. The updated Keynote‐048 report also provides additional subgroup analyses by age, sex, ECOG performance status, region of enrollment, smoking status, p16 status, and disease status [1, 12]. Although exploratory and not powered for definitive conclusions, these analyses suggest heterogeneity in treatment benefit. Hazard ratios generally favored pembrolizumab monotherapy over cetuximab chemotherapy, except in patients with recurrent‐only disease. Statistically significant benefits were observed in male patients (supplementary data in reference [1]), in those with p16‐negative oropharyngeal tumors, in patients with metastatic disease, and in participants enrolled outside Europe/North America [12]. Regional differences, however, should be interpreted with caution [13].

For CheckMate 141, two subgroup analyses have been published: one including 76 patients with R/M SCCHN who progressed on platinum therapy in the adjuvant or primary setting [14], and another including 62 patients who continued nivolumab beyond progression [15]. The updated two‐year data also reported subgroup outcomes by PD‐L1 status (with a significant OS benefit for PD‐L1 expressors, HR favoring nivolumab in non‐expressors) and HPV status (benefit in both HPV‐positive and HPV‐negative disease) [8].

These updates further support the robustness of the original trial results. At present, such subgroup findings can aid in identifying patients more likely to respond to immunotherapy, but they should not yet guide treatment decisions. Based on available evidence, the ideal candidate for pembrolizumab monotherapy appears to be a male patient with a high CPS, metastatic disease, and a p16‐negative oropharyngeal tumor.

The indications for pembrolizumab and nivolumab according to FDA approval overlap, so some patients are eligible for both drugs. Before choosing either pembrolizumab or nivolumab monotherapy, it should be evaluated if pembrolizumab + chemotherapy is a valid option for the patient since this treatment regimen drastically improves overall response rate and also shows longer PFS in the PD‐L1 ≥ 1% subgroup in comparison to nivolumab/pembrolizumab monotherapy and longer PFS in the PD‐L1 ≥ 20% subgroup in comparison to standard of care. This comes at the cost of a highly increased toxicity, and should therefore be carefully considered [1, 16]. Direct comparative data are limited, but a network meta‐analysis of the pivotal phase 3 trials found no significant difference in overall survival or progression‐free survival between the two drugs [17]. Another recent meta‐analysis comparing different immunotherapies in first and second line treatment of HNSCC found no significant difference in OS/PFS/ORR between nivolumab and pembrolizumab in first line treatment. In second line treatment, nivolumab showed significantly longer PFS in the PD‐L1 ≥ 1% subgroup when compared to pembrolizumab or standard of care, but no significant difference in OS when compared to pembrolizumab [16]. Real‐world data comparing the drugs directly are limited but report similar ORR and survival outcomes for both agents [18]. Since there are no clinically meaningful differences in efficacy, the choice between agents remains at the discretion of the attending physician and may be influenced by PD‐L1 status, toxicity profile, dosing schedule, or personal preference.

Among biomarkers, PD‐L1 expression remains the only established predictor of OS in HNSCC, and its predictive value has been validated only for pembrolizumab [1]. Subgroup analyses by CPS highlight the need for better predictors, particularly in the CPS < 1 group, as many patients with low CPS still derive benefit from ICI therapy [8, 19] and PD‐L1 expression does not seem to correlate with the disease control rate [4] Other studies have identified markers that can predict outcomes in patients treated with ICI, but most concentrate on solid tumors in general, melanoma, or non‐small‐cell lung cancer (NSCLC) [3, 20]. While tumor mutational burden (TMB) is a reliable predictor of response in ICI therapy for other cancers, its predictive value in HNSCC appears to be limited to HPV‐negative tumors, and TMB is not routinely assessed in HNSCC patients [21].

Our data identified the absence of alcohol and tobacco abuse and a TPS ≥ 50% as predictors of response to ICI therapy. Notably, a high CPS score only influenced PFS in the univariate analysis in our cohort, without significance in any multivariate analysis, which contrasts with the Keynote‐048 findings [1]. Some other real‐world data reported similar outcomes [9, 22] which might be due to the small data set. Previous studies have shown that smoking alone is not a reliable predictor of response in HNSCC [21]. Interestingly, while studies in NSCLC have reported higher response rates among smokers, possibly due to higher TMB [23], our findings suggest that the absence of both smoking and alcohol consumption is associated with improved response rates in HNSCC. Mechanistically, smoking may increase TMB and PD‐L1 expression and thereby enhance ICI efficacy in NSCLC, but in HNSCC, it appears to exert predominantly immunosuppressive effects [24]. In contrast, chronic alcohol consumption impairs both innate and adaptive immunity, interfering with T‐cell signaling and cytokine production, which may diminish antitumor immune responses [25, 26]. This stresses the significance of educating patients not only about the role of smoking and alcohol abuse in tumorigenesis but also in treatment efficacy and informing them about addiction support and counseling [27]. Patients are often conscious of the risks of smoking but are not informed enough about the risks of even moderate alcohol consumption [28, 29].

We also collected data on immune‐related adverse events within our cohort. Previous studies suggest that irAEs in HNSCC and other tumor entities are associated with improved oncologic outcomes [22, 30, 31]. In our cohort, irAEs of grade 2 or higher were associated with longer OS and PFS, although this reached significance only in the univariate analyses. This is likely attributable to the small number of patients (*n* = 10, 22%) reporting irAEs. In comparison, the reported frequency of irAEs in the literature is usually around 50%–60% [1, 2]. Among these patients, eight did not experience disease progression, and notably, all patients who discontinued treatment due to irAEs remained progression‐free over 12 months after treatment discontinuation. This finding aligns with previous reports of the lasting effect of ICI even after discontinuing treatment [12]. IrAEs are often underreported without explicit monitoring, and the growing experience in recognizing and managing irAEs underscores the importance of systematic assessments to better understand their association with clinical outcomes [32].

Our analysis of patient‐specific factors revealed that female sex (only in univariate analysis for OS), NLR > 5, and a leukocyte count above the norm were significantly associated with worse OS and PFS, while ORR was positively associated with OS and PFS. Female sex was significantly associated with worse survival in the univariate analysis but narrowly missed statistical significance in the multivariate analysis. Other factors that were only significant in univariate analyses have been described to have a significant negative effect on OS/PFS in other studies (primary in the oral cavity [10], high ECOG [9, 10], high GPS [33]). It is well‐established that patients responding to ICI therapy generally experience better OS and PFS than non‐responders. However, the finding that female sex is a negative predictor of OS and PFS is novel. Most studies, including Keynote‐048 and CheckMate 141, include predominantly male patients. Our cohort, with 24% female patients, suggests that women benefit less from ICIs. A recent meta‐analysis supports this significant difference in the efficacy of immunotherapy between men and women across various tumors [34] including a specific subset analysis for Keynote‐048 and CheckMate 141. Subgroup analyses in the supplemental data of the Keynote‐048 study also suggest that female patients benefit less from immunotherapy than male patients [1].

Abnormal leukocyte counts and their subtypes have been proposed as a predictive marker for immunotherapy outcomes in other studies. The most frequently cited parameter in HNSCC is the NLR. Although no universally accepted cut‐off exists, the most commonly reported thresholds range between 4 and 7 [4]. A high NLR is consistently associated with worse OS and PFS [35, 36, 37], which we confirmed in our cohort. Most studies include only melanoma or NSCLC [3] and suggest an association between elevated lymphocytes and improved OS. An elevated lymphocyte count has been shown to improve OS in HNSCC treated with ICI, and a low lymphocyte count is thought to worsen ORR in HNSCC [9, 37]. Lymphocyte count alone did not have a significant effect on ORR/PFS or OS in our study. We categorized the lymphocyte count as within the normal range (1.4–3.2/nL) versus below or above this range. Other studies have defined cut‐off values for absolute lymphocyte count at 0.6 or 0.9/nL, which are therefore much lower than in our cohort, and have demonstrated an association between low lymphocyte count and shorter PFS as well as poorer response [37]. One study associated a prolonged OS with a leukocyte count below the upper limit of normal, which is in line with our findings [38].

A recent meta‐analysis in patients with HNSCC treated with immunotherapy evaluated the prognostic impact of HPV status, PD‐L1 expression, and a range of laboratory parameters. For OS, longer survival was observed in patients with PD‐L1 expression, HPV positivity, lower GPS grading, lower ECOG performance status, higher BMI, lower NLR, lower PLR, higher albumin levels, and lower LDH. Prolonged PFS was correlated with PD‐L1 expression, HPV positivity, lower GPS grading, lower performance status, higher BMI, lower NLR, higher absolute lymphocyte count, and lower LDH [4]. In our study, univariate analysis also showed an effect of GPS, ECOG score, and albumin on OS and PFS, but these results were not confirmed in the multivariate analysis. The association between low NLR and longer OS/PFS was confirmed in our cohort. HPV status did not have an effect on OS in our study, which might be due to the small sample size. HPV positivity and PD‐L1 expression were both associated with improved response [4]; however, in our cohort, this association was observed only for PD‐L1 expression. Interestingly, HPV positivity and PD‐L1 expression were not associated with disease control rate. The abovementioned meta‐analysis included all immunotherapies and combination therapies without subgroup analyses for pembrolizumab or nivolumab.

In conclusion, our study demonstrates that real‐world outcomes in HNSCC patients treated with ICI are comparable to those reported in the Keynote‐048 and CheckMate 141 studies. We confirmed that ORR is predicted by a TPS ≥ 50% and showed that patients without a history of smoking or alcohol consumption are more likely to respond to ICIs. OS and PFS were significantly improved in patients with a positive treatment response but were worsened in female patients and those with elevated leukocyte counts and high NLR. Future studies on immunotherapy in HNSCC should include a higher proportion of female patients to better understand sex‐specific differences and avoid the inappropriate generalization of findings predominantly derived from male cohorts. Larger cohorts will help to confirm our findings and clarify the differences that were not significant in our study.

### 4.1 Limitations of the Study

This study was conducted with a small sample size and at a single center to help guide the direction of future research. Consequently, the statistical power is limited, and the findings need to be confirmed in larger, multi‐center cohorts. Such studies would also allow for a clearer distinction between pembrolizumab and nivolumab, both of which are approved for R/M HNSCC, albeit in slightly different clinical settings. Given that HNSCC represents a heterogeneous group of tumors, it would be preferable to either focus on a single tumor site or perform subgroup analyses—neither of which was feasible here due to the small sample size. Although the pivotal trials Keynote‐048 and CheckMate 141 included multiple tumor sites and CheckMate 141 reported tumor site‐specific subgroup analyses, even these large studies were not powered for definitive site‐specific comparisons. The subgroup analyses in the CheckMate 141 study showed similar hazard ratios across all tumor sites.

## Author Contributions

**Lena Huber:** writing – original draft, writing – review and editing, conceptualization, visualization. **Constanze Kohler:** investigation, methodology, formal analysis, data curation. **Anne Lammert:** supervision. **Claudia Scherl:** supervision. **Sonja Ludwig:** supervision. **Annette Affolter:** project administration, resources. **Nicole Rotter:** supervision. **Frederic Jungbauer:** writing – review and editing, validation.

## Ethics Statement

This retrospective single‐center study was approved by the ethics committee of the University of Heidelberg (2024‐807).

## Consent

No case details, personal information or images are included. Individuals cannot be identified. The ethics committee of the University of Heidelberg did therefore not require patient consent for this retrospective study.

## Conflicts of Interest

The authors declare no conflicts of interest.

## Data Availability

The data that support the findings of this study are available from the corresponding author upon reasonable request.
